# Equivalence of difluorodichloromethane (CFC-12) hydrolysis catalyzed by solid acid(base) MoO_3_(MgO)/ZrO_2_

**DOI:** 10.1039/d0ra05947a

**Published:** 2020-09-11

**Authors:** Zhiqian Li, Xiaofang Tan, Guoqin Ren, Yu Chang, Lijuan Jia, Kaijiao Duan, Tiancheng Liu

**Affiliations:** a College of Chemistry and Environment, Yunnan Minzu University, National and Local Joint Engineering Research Center for Green Preparation Technology of Biobased Materials Kunming 650500 Yunnan China liutiancheng76@163.com +8613708893755; b Yunnan Technician College Kunming Yunnan 650500 China

## Abstract

In this paper, a solid acid(base) MoO_3_(MgO)/ZrO_2_ was prepared for the catalytic hydrolysis of difluorodichloromethane (CFC-12). The effects of the catalyst preparation method, calcination temperature, and hydrolysis temperature on the conversion rate of CFC-12 were studied. The catalysts were characterized by XRD, N_2_ isotherm adsorption desorption, NH_3_-TPD, and CO_2_-TPD. Meanwhile, the equivalence of the catalytic activity of MoO_3_(MgO)/ZrO_2_ for CFC-12 was studied. Research shows that the solid acid MoO_3_/ZrO_2_ and solid base MgO/ZrO_2_ catalyzed hydrolysis of CFC-12 is equivalent; the solid acid MoO_3_/ZrO_2_ is calcined at 600 °C for 3 h and the solid base MgO/ZrO_2_ is calcined at 600 °C for 6 h (co-precipitation) and 700 °C for 6 h (impregnated) at a catalytic hydrolysis temperature of 300–400 °C and CFC-12 concentration of 4%. The catalytic hydrolysis products obtained were CO, HCl, and HF, and the CFC-12 conversion rate almost reached 100%.

## Abstract 2

In this paper, a solid acid(base) MoO_3_(MgO)/ZrO_2_ was prepared for the catalytic hydrolysis of difluorodichloromethane (CFC-12).

## Graphical abstract

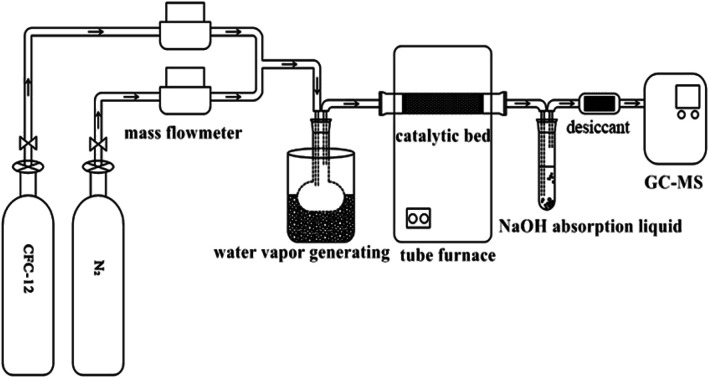

## 1 Introduction

Chlorofluorocarbons (CFCs) are a refrigerant trademark registered by the DuPont company in the United States. They were first invented in 1928 by Midgley (Thomas, Jr) in the United States. In 1980s, CFC-12 was widely used in various fields of production and life due to its excellent physical and chemical properties and low production costs, such as cleaning solvents, refrigerants, thermal insulation materials, sprays, and foaming agents,^1^ and its output had reached 1.44 million tons. Before the output could be controlled, the amount of CFC-12 emission had reached 20 million tons in the world.^2^ However, in June 1974, professor Rowland and Dr Molina of the University of California published a paper describing that the chlorine radicals produced by CFC decomposition in the stratosphere can rapidly decompose ozone and lead to the ozone hole.^3^ CFCs are also greenhouse gases,^4^ with a greenhouse effect of 3400–15000 times that of CO_2_ and 300–1400 times that of CH_4_; the emission of CFCs in the atmosphere leads to the increase in greenhouse gases on the earth, which leads to global warming,^5^ melting of polar glaciers, and rise in the sea level with serious adverse effects on the global environment and ecological balance.^6–9^ At present, the harmless treatment technologies of CFC-12 mainly include high temperature destruction method,^10^ plasma method,^11–13^ ray decomposition method,^14,15^ electrochemical decomposition method,^16–18^ and catalytic hydrogenation methods.^19–21^ However, these methods have certain limitations. Thus, it is urgent to find a safe and efficient method to degrade CFC-12. As a practical technology, the catalytic hydrolysis method has been studied more in the recent years. The catalytic hydrolysis method has the following advantages: it is easy to carry out thermodynamically; the required decomposition temperature is lower than that of other methods; the necessary reactants include water vapor, which is easily available; the catalytic hydrolysis process is simple and easy to set up; the catalytic hydrolysis products are HF and HCl that are easily neutralized by an alkali solution to avoid secondary pollution; other secondary hazardous waste is not generated, such as dioxin and fly ash produced by incineration.

In view of the environmental pollution caused by a large number of low concentration CFC-12 gas^22^ discharged into the atmosphere, the technology of catalytic hydrolysis of CFC-12 with solid acid and solid base catalyst is proposed, and equivalence analysis is carried out. As an example, CFC-12 was mixed with water vapor and nitrogen was used as the equilibrium gas. After passing through the catalytic reaction bed filled with the catalyst, it can achieve the catalytic hydrolysis of low concentration CFC-12. The highest catalytic hydrolysis was 99.64%, in which Freon was almost completely degraded. This research project provides a certain basis for the harmless treatment of low concentration CFC-12. At the same time, it has a very important guiding and practical significance for promoting the technical field of solving CFC-12 and other CFCs in China.

## 2 Experimental

### 2.1. Materials and reagents

Gas CFC-12, Zhejiang Ju Hua limited company; N_2_, Kunming Guang rui da Gas Company Limited; ZrOCl_2_·8H_2_O (A. R.), Sino pharm group Chemical Reagents Company Limited; Mg(NO_3_)_2_·6H_2_O (A. R.), (NH_4_)_6_Mo_7_O_24_·4H_2_O (A. R.), SiO_2_ (A. R.), Tianjin wind boat Chemical Reagents Company Limited; NH_3_·OH (25%) (A. R.), AgNO_3_ (A. R.), Guangdong Guanghua Technology limited company; NaOH (A.R.), Chengdu Jinshan Chemical Reagents Company Limited.

### 2.2. Experimental process

**Fig. 1:**
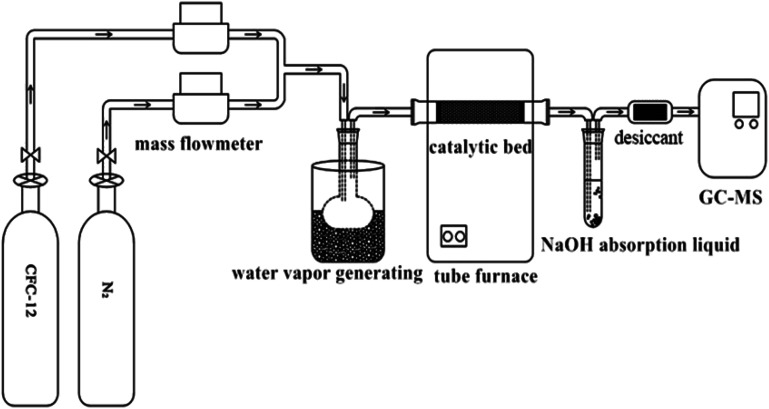
Flow diagram of the CFC-12 catalytic decomposition experiment.

Quartz sand (the main component is SiO_2_) was used as a catalyst filler carrier, and 1.00 g of catalyst and 50 g of quartz sand were evenly mixed and filled in the quartz tube. Simulated reaction gas composition (mol%): 4.0 CFC-12, 25.0H_2_O(g), 5% O_2_, and the rest is N_2_. The generated acid gases HCl and HF were absorbed with an alkaline solution (NaOH solution) and silica gel was used as a drying agent. Sampling was carried out 10 min after reaching the required reaction conditions, and the collected gas was qualitatively and quantitatively analyzed by gas chromatography-mass spectrometry (Fig. 1).

### 2.3. Catalyst characterization

The phase composition of the sample was measured by a Bruker D8 Advance X-ray diffractometer in Germany under the following conditions: Cu target, Kα radiation source, 2*θ* range of 10–90°, scanning rate of 12° min^−1^, step length of 0.01° s^−1^, working voltage and current of 40 kV and 40 mA, *λ* = 0.154178 nm. The specific surface area was calculated using the Brunauer–Emmett–Teller (BET) equation from N_2_ physisorption at −196 °C. The pore size distribution was measured by the BJH method from the adsorption branch. The surface acidity and basicity of the sample were measured by a Das-7200 automatic chemical adsorption instrument. 0.1 g sample was put into a quartz U-shaped tube, pretreated in nitrogen (30 mL min^−1^) at 200 °C for 1 h, cooled to 50 °C, absorbed in ammonia (30 mL min^−1^) at this temperature for 0.5 h, switched to He gas for purging until the signal was stable, heated to 900 °C at the rate of 10 °C min^−1^, and detected by the TCD-NH_3_ desorption signal. After pretreatment for 2 h at 700 °C in an argon atmosphere, the impurities on the surface were removed. When it was cooled to 30 °C, CO_2_ was absorbed to saturation. Then, CO_2_ on the surface was purged for 0.5 h by argon. Finally, the temperature was raised to 900 °C at the rate of 10 °C min^−1^. The CO_2_ desorption signal was detected by TCD.

### 2.4. Analytical method

Gas chromatography-mass spectrometry (GC/MS) was used for quantitative and qualitative analysis. The instrument was Thermo Fisher ISQ manufactured by Thermo-Fisher Scientific, Inc. The column was a capillary column (100% dimethylpolysiloxane) manufactured by Thermo Fisher Scientific; the detection conditions were: inlet temperature 80 °C, column temperature 35 °C, retention time 3 min, carrier gas was high purity He (He ≥ 99.999%), flow rate of 1.00 mL min^−1^ in constant flow mode, split ratio was 100 : 1. The mass spectrometer detector was an EI source with an electron energy of 70 eV, an ion source temperature of 260 °C, an ion transport rod temperature of 280 °C, and an injection volume of 0.1 mL.^23^ The qualitative and quantitative analyses of CFC-12 were performed under this analysis condition. The catalytic decomposition effect was mainly evaluated by the conversion rate of CFC-12 and the total yield of CO and CO_2_, and the calculation was as follows[Conversion rate of CFC-12] = ([CFC-12]_in_ − [CFC-12]_out_)/[CFC-12]_in_ × 100%;[Yield of CO_*x*_] = [CO_*x*_]_out_/([CFC-12]_in_ − [CFC-12]_out_) × 100%

## 3 Results and discussion

### 3.1. Preparation of the MoO_3_/ZrO_2_ solid acid catalyst

The catalyst was prepared by the impregnation method. Firstly, 0.15 mol L^−1^ ZrOCl_2_·8H_2_O solution was placed in a 250 mL beaker. The water bath was heated to 60 °C and stirred to dissolve the salt. Then, 25% ammonia solution was slowly added until the pH value reached 9–10. It was then continually stirred at 60 °C for 1 h. Next, it was allowed to stand at room temperature for 12 h and washed until free from Cl^−^ (detected with 0.1 mol L^−1^ AgNO_3_ solution), and the resulting filter cake was dried at 110 °C for 12 h. Then, it was ground and impregnated with 0.5 mol L^−1^ (NH_4_)_6_Mo_7_O_24_·4H_2_O solution for 4 h (ZrO_2_ mass fraction is 20%) at 80 °C, followed by filtration, drying at 110 °C for 24 h, and calcination at 500 °C, 550 °C, 600 °C, 650 °C, and 700 °C for 3 h. The obtained samples were ground to obtain the MoO_3_/ZrO_2_ catalyst.

### 3.2. Characterization of the MoO_3_/ZrO_2_ solid acid catalyst

#### 3.2.1. XRD patterns of MoO_3_/ZrO_2_ calcined at different temperatures

**Fig. 2:**
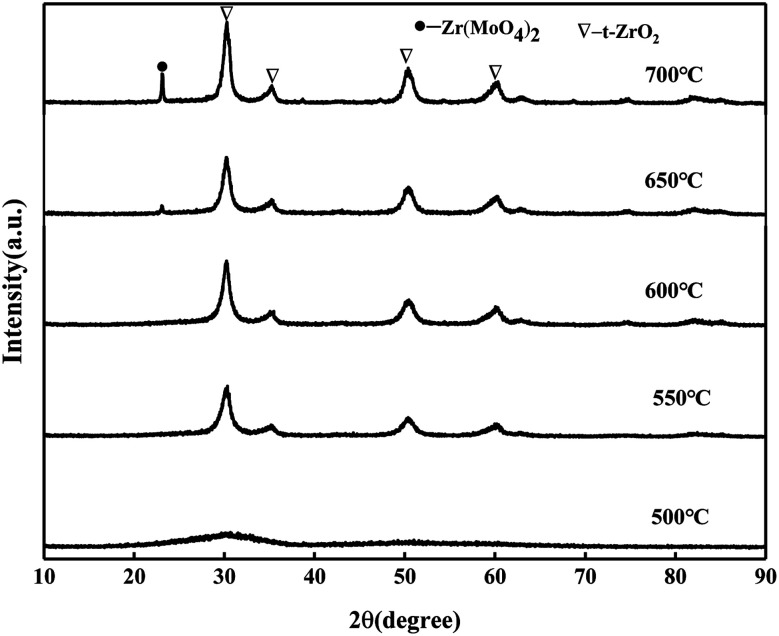
XRD patterns of MoO_3_/ZrO_2_ calcined at different temperatures.

It can be seen from Fig. 2 that the catalyst samples calcined at 550 °C and 600 °C have obvious characteristic peaks at 30.3°, 35.3°, 50.4°, and 60.3° belonging to the tetragonal phase of ZrO_2_. The intensity of the diffraction peaks increases with the increase in the calcination temperature, indicating that the crystallization degree is further improved. When the calcination temperature is 650 °C and 700 °C, the characteristic peak of the Zr(MoO_4_)_2_ phase at 2*θ* = 23.1° was slowly enhanced with the increase in the calcination temperature due to the reaction between MoO_3_ and Zr(OH)_4_ to form Zr(MoO_4_)_2_.^24^ The diffraction peak of MoO_3_ was not detected, indicating that MoO_3_ in the catalyst was highly dispersed on the surface of ZrO_2_ or permeated into the framework of ZrO_2_ in an amorphous state.^25^

#### 3.2.2. TEM characterization of MoO_3_/ZrO_2_

**Fig. 3:**
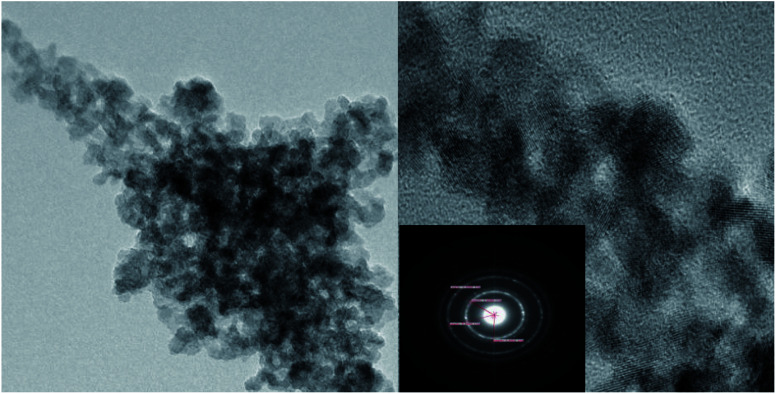
TEM characterization of the MoO_3_/ZrO_2_ solid acid calcined at 600 °C for 3 h.

From the TEM diagram of MoO_3_/ZrO_2_, it can be seen that MoO_3_ and ZrO_2_ have an obvious interaction. When the calcination temperature is 600 °C and the calcination time is 3 h, the catalyst prepared by the impregnation method mainly exists in the crystal form. The selected area diffraction pattern shows a concentric circle, which shows that the sample is polycrystalline, which is consistent with the XRD results (Fig. 3).

#### 3.2.3. N_2_ adsorption–desorption characterization of MoO_3_/ZrO_2_

**Fig. 4:**
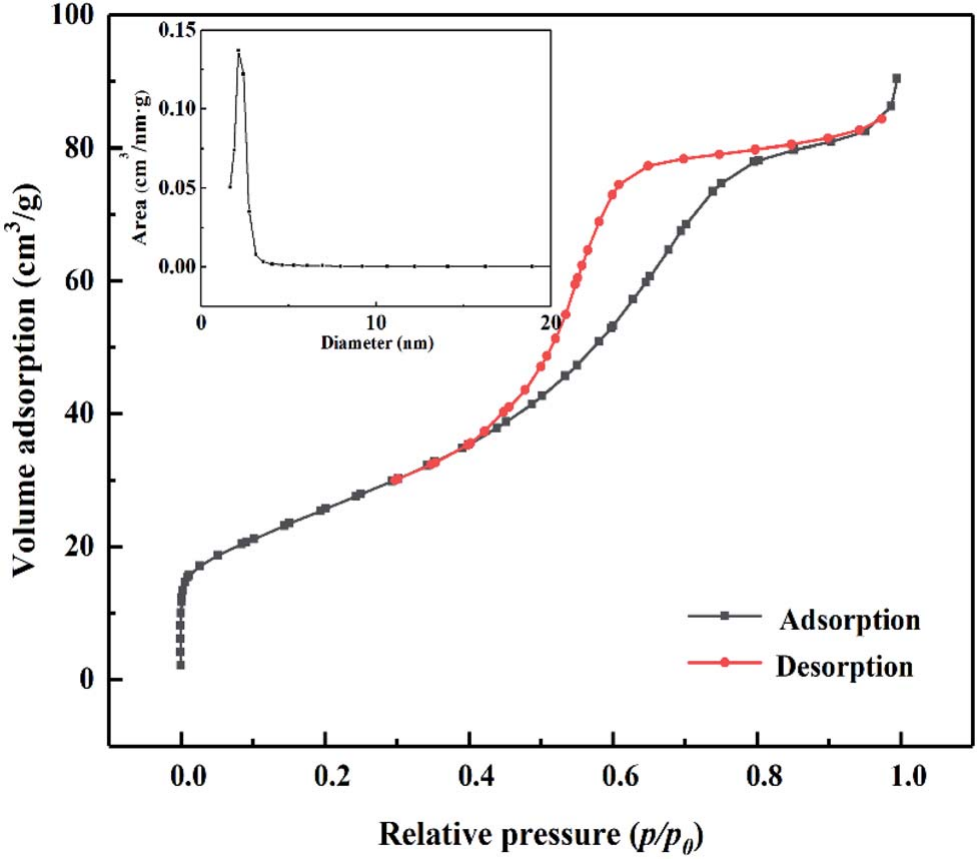
N_2_ adsorption desorption curve of MoO_3_/ZrO_2_ heat-treated at 600 °C.

As shown in Fig. 4, according to the Brunauer, Deming, Deming, and Teiler (BDDT) classification, the adsorption desorption isotherm of the MoO_3_/ZrO_2_ catalyst belongs to class IV, and the adsorption hysteresis phenomenon is shown in the figure. The generation of the hysteresis ring H_2_ is caused by a porous adsorbate and uniform particle accumulation pore, indicating that the solid particles in the composite material have a mesoporous structure. In the low-pressure zone, the adsorption–desorption isotherm curve deviates from the *y*-axis, which indicates that the catalyst and nitrogen have a strong interaction and that there are many micropores in the catalyst. In the middle pressure zone, the multi-layer adsorption of the catalyst gradually occurs, the adsorption capacity sharply increases, the adsorbate condenses in the capillary, and the isotherm rises. At this time, the adsorption isotherm and desorption isotherm do not coincide, and for the desorption isotherm position, the change in the slope of the isotherm is high when the relative pressure is 0.4, which indicates that the homogeneity of the mesoporous materials is good. Also, when the relative pressure is close to 1.0, the curve rises, which indicates that the sample may have particle accumulation or a macroporous structure.

#### 3.2.4. NH_3_ temperature-programmed desorption (NH_3_-TPD)

**Fig. 5:**
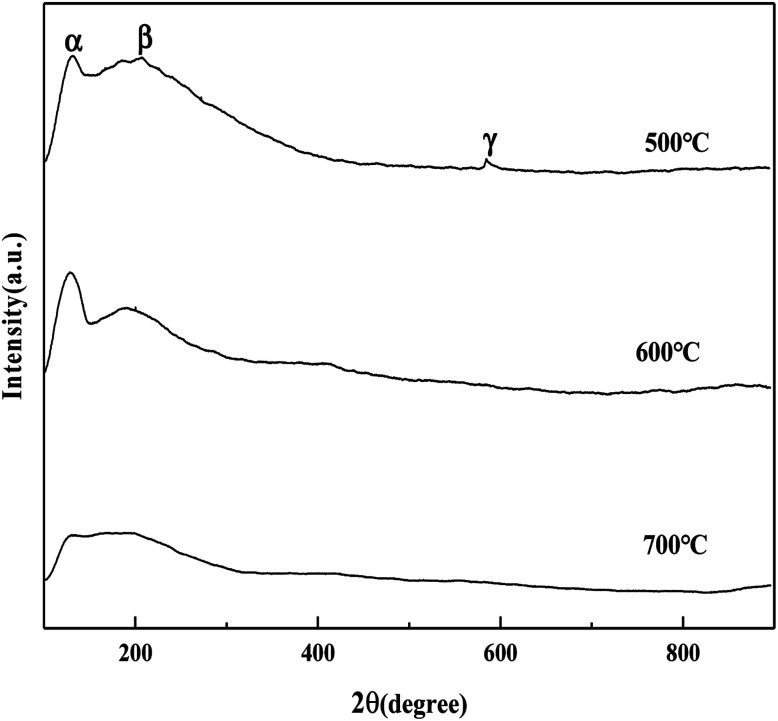
NH_3_ temperature-programmed desorption of MoO_3_/ZrO_2_.

It can be seen from Fig. 5 that the MoO_3_/ZrO_2_ catalyst calcined at 500 °C has three NH_3_ desorption peaks (α, β, and γ), which correspond to the weak acid site desorption peak, the medium-strong acid site desorption peak, and the strong acid site desorption peak.^26^ NH_3_ desorption temperature is in the range of 100–150 °C, 200–230 °C, and 500–700 °C; the size of the peak area is proportional to the amount of NH_3_ adsorbed by the sample, which is proportional to the amount of acid in the sample, and the γ desorption peak is weak. It shows that its strong acid content is less and the α desorption peak of the MoO_3_/ZrO_2_ catalyst calcined at 600 °C is the strongest, indicating that its weak acid content is the largest. The α desorption peak of the MoO_3_/ZrO_2_ catalyst calcined at 700 °C is the weakest, indicating that its weak acid content is the least; previous experimental studies have shown that the MoO_3_/ZrO_2_ catalyst calcined at 600 °C has the best effect on the hydrolysis of CFC-12 and the worst at 700 °C. Combined with the NH_3_-TPD characterization results, it shows that when the solid acid MoO_3_/ZrO_2_ catalyzes the hydrolysis of CFC-12, the weak acid site also has a strong catalytic activity. From the characterization results of NH_3_-TPD, it can also be seen that the calcination temperature has a great influence on the catalyst. The γ desorption peaks of the MoO_3_/ZrO_2_ catalysts calcined at 600 °C and 700 °C disappeared. Thus, high calcination temperature is not conducive for the formation of strong acid sites.

#### 3.2.5. XPS characterization of the solid acid MoO_3_/ZrO_2_ before and after reaction

**Fig. 6:**
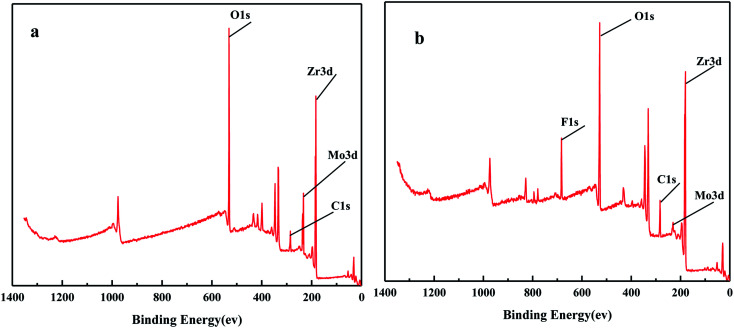
The full XPS spectrum of the solid acid MoO_3_/ZrO_2_.

**Fig. 7:**
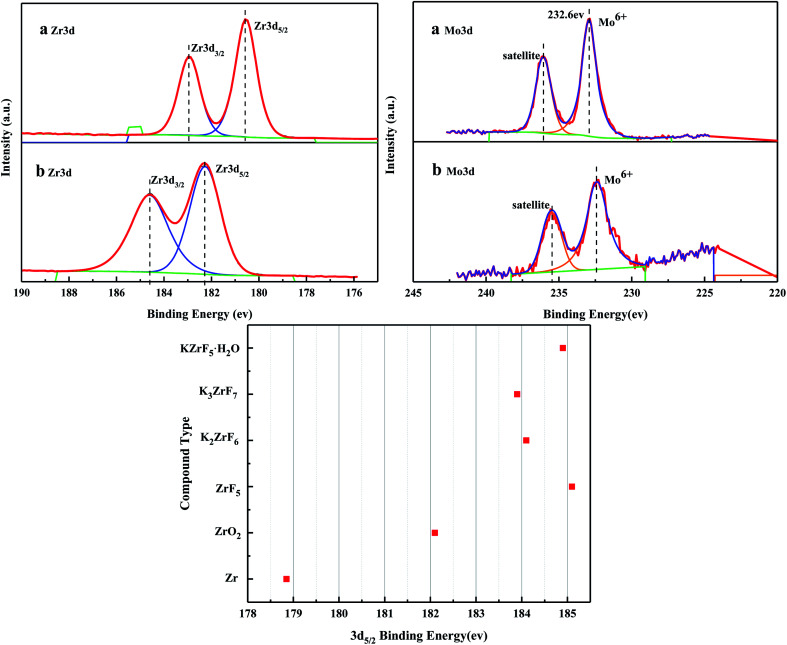
XPS fitting spectra of solid acid MoO_3_/ZrO_2_ before (a) and after (b) the reaction.

Fig. 6 shows the XPS spectra before and after the MoO_3_/ZrO_2_ reaction. It can be seen from the figure that F appears in the catalyst after the reaction mainly due to the introduction of CFC-12. The spectra of MoO_3_/ZrO_2_ before and after the reaction were fitted. In the Zr 3d spectrum in Fig. 7, there are two main peaks at 179.05 eV and 182.49 eV, as found out by consulting the Handbook of X-ray photoelectron spectroscopy, which belong to the Zr and Zr^4+^ species. From the XPS parameters in Table 1, it can be seen that the content of Zr decreases after the reaction, while the content of Zr^4+^ increases, indicating that Zr reacts in the oxidation reaction. For the Mo 3d spectrum, there is a main peak at 232.6 eV, which confirms the existence of MoO_3_, as can be seen from the X-ray photoelectron spectroscopy manual. According to the XPS parameters in Table 1, the amount of MoO_3_ decreased after the reaction, indicating the reduction of Mo^6+^.

**Table 1:** XPS characterization of solid acid MoO_3_/ZrO_2_ before and after the reaction

Name	Peak BE	Areas (*P*) CPS. eV
a Zr3d_5/2_	179.05	91 551.47
Zr3d_3/2_	182.5	60 087.61
b Zr3d_5/2_	182.41	79 567.05
Zr3d_3/2_	1843	69 248.59
a Mo3d	232.9	35 922.37
b Mo3d	232.50	6448.93

#### 3.2.6. Effects of catalytic hydrolysis temperature and calcination temperature of MoO_3_/ZrO_2_ on catalytic hydrolysis

**Fig. 8:**
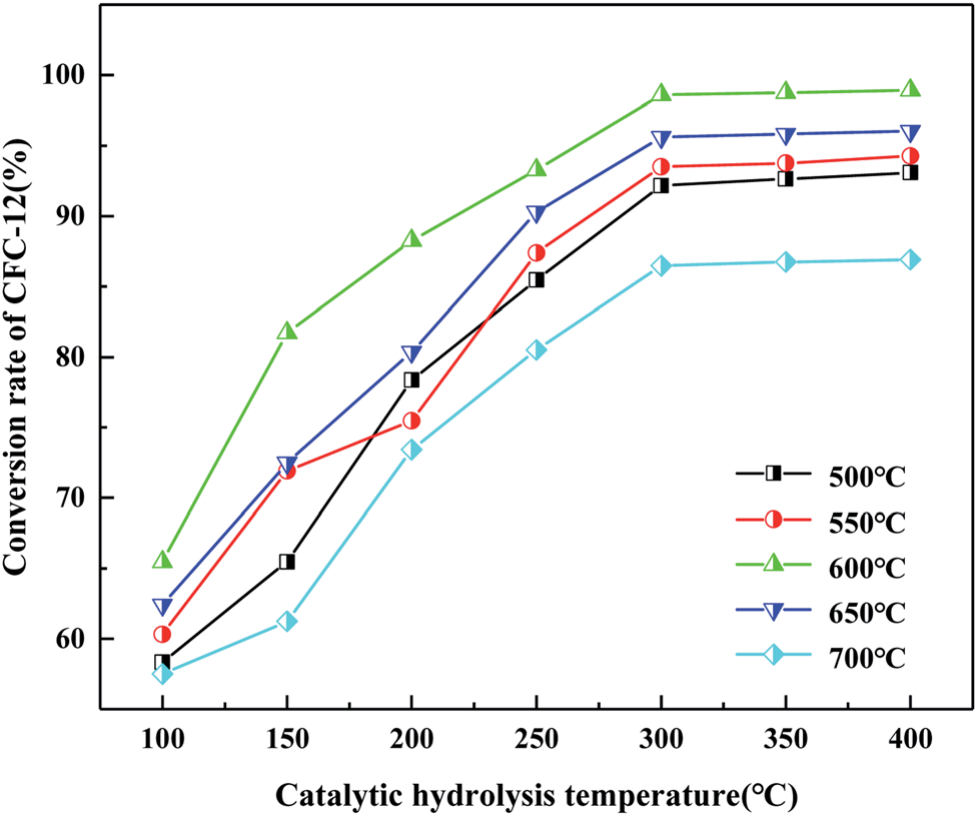
Effect of calcination temperature and catalytic hydrolysis temperature on the hydrolysis rate.

It can be seen from Fig. 8 that the hydrolysis rate is 98.91% when the calcination temperature of MoO_3_/ZrO_2_ is 600 °C and the catalytic hydrolysis temperature is 300 °C. MoO_3_/ZrO_2_ has a moderate specific surface area and pore size when the calcination temperature is 600 °C, and the content of weak acid is high, which is conducive for improving the catalytic activity of the catalyst. When the calcination temperature of MoO_3_/ZrO_2_ was 700 °C, the conversion rate of CFC-12 was only 86.91%. The main reason is that when the calcination temperature is too high, the catalyst will be sintered, which will destroy the surface structure of the catalyst, thus reducing the catalytic activity and the hydrolysis rate.

#### 3.2.7. Comparison of catalytic hydrolysis of CFC-12 by MoO_3_, ZrO_2_, and MoO_3_/ZrO_2_

**Fig. 9:**
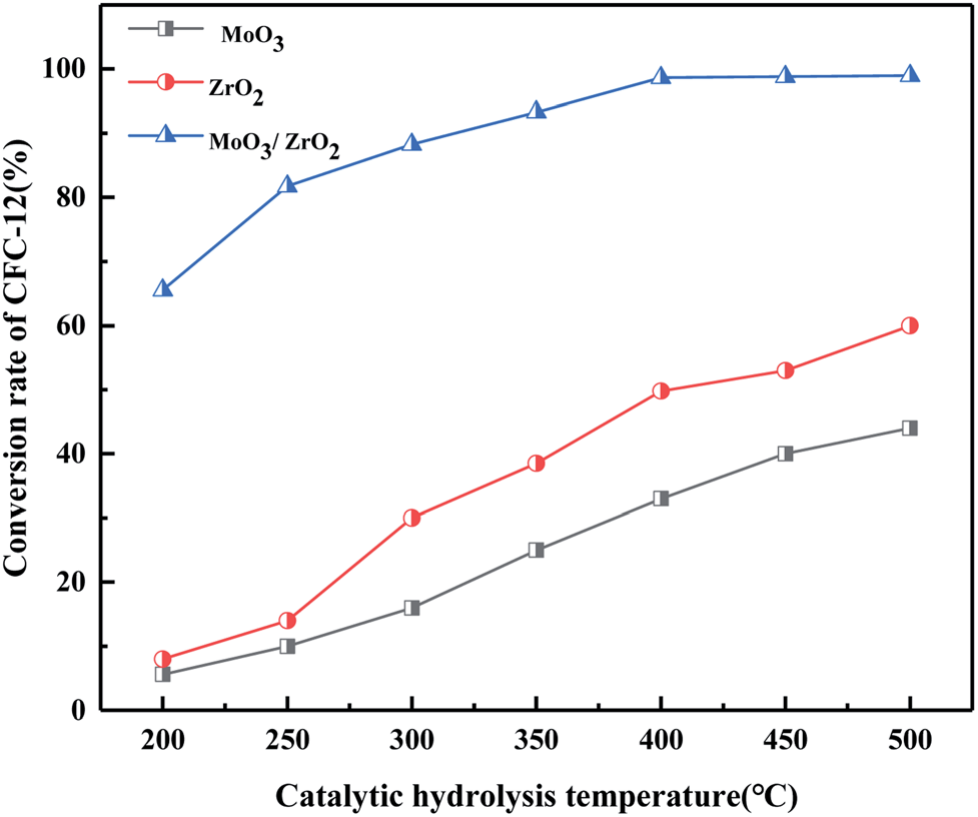
Comparison of catalytic hydrolysis of CFC-12 by MoO_3_, ZrO_2_, and MoO_3_/ZrO_2_.

It can be seen from Fig. 9 that both MoO_3_ and ZrO_2_ have a certain activity for the catalytic decomposition of CFC-12, and ZrO_2_ has a higher catalytic activity than that of MoO_3_. The conversion of CFC-12 increases with the increase in the catalytic hydrolysis temperature. The conversion of CFC-12 by MoO_3_ and ZrO_2_ was 40.12% and 59.63% at 500 °C; the MoO_3_/ZrO_2_ solid superacid has higher catalytic activity and the conversion of CFC-12 reached 98.91% at 400 °C. The comparative experiments show that MoO_3_/ZrO_2_ is more efficient than MoO_3_ and ZrO_2_ in the degradation of CFC-12.

### 3.3. Preparation of MgO/ZrO_2_ solid base catalyst by co-precipitation and impregnation method

ZrOCl_2_·8H_2_O and Mg (NO_3_)_2_·6H_2_O were used to prepare a 0.15 mol L^−1^ aqueous solution with *n*(Mg) : *n*(Zr) = 0.3 : 1 by the co-precipitation method. The water bath was heated to 60 °C and stirred to dissolve the salts. 25% aqueous ammonia solution was slowly added until the pH = 9–10, stirred continually at 60 °C for 1 h, then stood at room temperature for 12 h, followed by washing until free from Cl^−^ (detected with 0.1 mol L^−1^ AgNO_3_ solution). The resulting filter cake was dried at 110 °C for 12 h. They were calcined at 500 °C, 600 °C, 700 °C, and 800 °C for 6 h, and ground to obtain the MgO/ZrO_2_ catalyst.

0.15 mol L^−1^ ZrOCl_2_·8H_2_O solution was prepared by the impregnation method in a 250 mL beaker. The water bath was heated to 60 °C and stirred to dissolve the salt. 25% ammonia solution was slowly added until the pH = 9–10, stirred for 1 h, then stood at room temperature for 12 h, and washed until there was no Cl^−^ (detected with 0.1 mol L^−1^ AgNO_3_ solution). The resulting filter cake was dried at 110 °C for 12 h. The dried filter cake was ground, immersed in 0.5 mol L^−1^ Mg (NO_3_)_2_·6H_2_O solution for 12 h (*n*(Mg) : *n*(Zr) = 0.3 : 1), immersed at 40 °C, and filtered. The filter cake was dried at 110 °C for 12 h, calcined at 500 °C, 600 °C, 700 °C, and 800 °C for 6 h, and ground to obtain the MgO/ZrO_2_ catalyst.

### 3.4. Characterization of the MgO/ZrO_2_ solid acid catalyst

#### 3.4.1. XRD of MgO/ZrO_2_ prepared by the co-precipitation and impregnation method

**Fig. 10:**
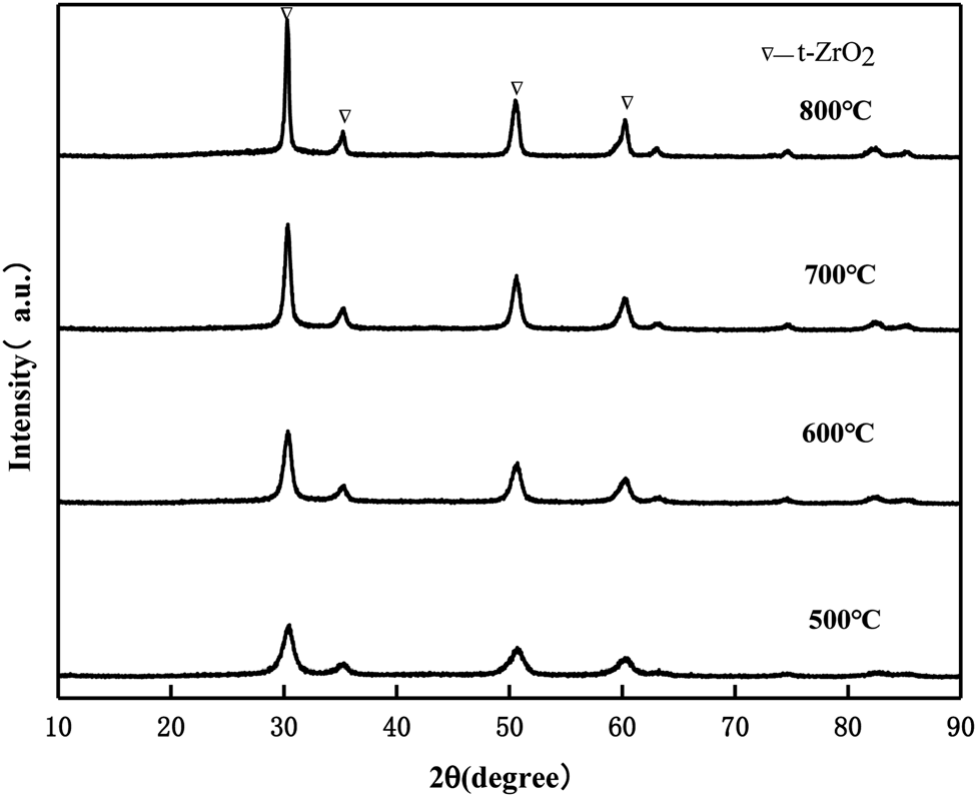
XRD pattern of MgO/ZrO_2_ prepared by the co-precipitation method.

It can be seen from Fig. 10 that the crystal phase of ZrO_2_ exists in the form of tetragonal phase in MgO/ZrO_2_ prepared by the co-precipitation method and the diffraction peak of MgO was not found, which indicates that MgO exists in the amorphous form. By the co-precipitation method, Mg (NO_3_)_2_·6H_2_O is added and Mg^2+^ enters the ZrO_2_ crystal lattice to replace the Zr^4+^ position to form the MgO/ZrO_2_ solid solution. With the increase in the calcination temperature, the diffraction peak of ZrO_2_ becomes sharper and the enhancement in the diffraction peak is weaker. It can be seen that the surface sintering degree of the catalyst is weaker. With the increase in the calcination temperature, the specific surface area of the catalyst gradually decreases, which is consistent with the BET characterization results.

**Fig. 11:**
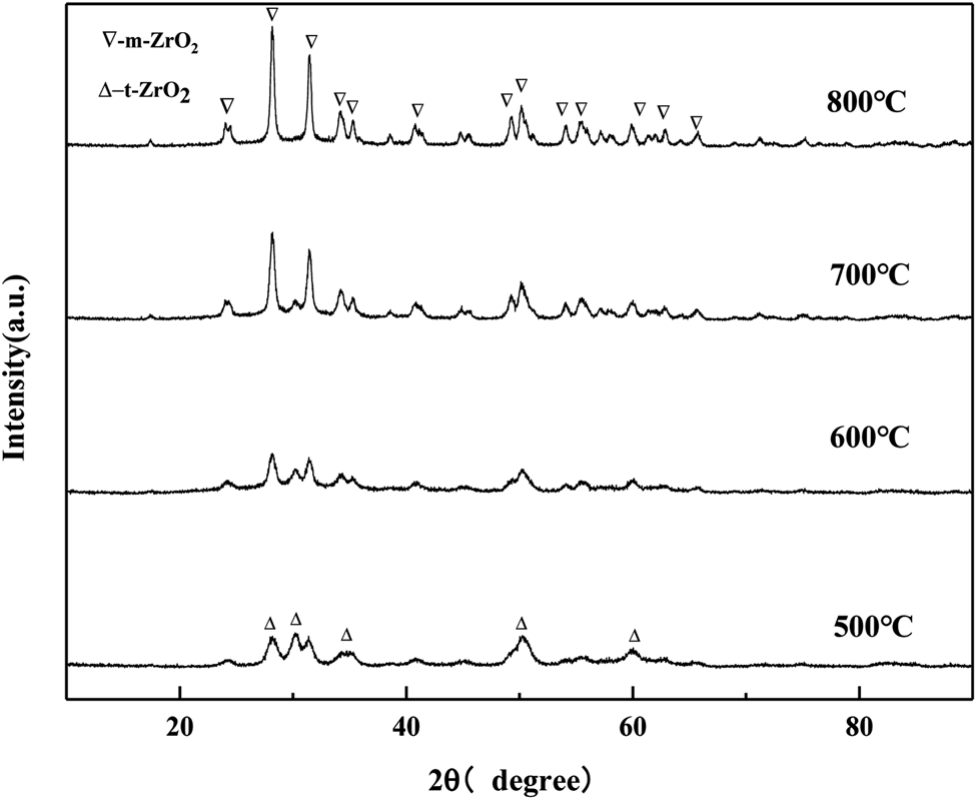
XRD pattern of MgO/ZrO_2_ prepared by the impregnation method.

It can be seen from the Fig. 11 that when the calcination temperature is 500 °C, the crystal phase of ZrO_2_ is a mixed state of the tetragonal phase and the monoclinic phase, and its diffraction peak intensity is relatively weak. The monoclinic phase ZrO_2_ gradually protruded, and the diffraction peak becomes more and more sharp, indicating that the grain size of the catalyst samples increases after sintering to a certain extent, which is consistent with the BET characterization results of the catalyst. The diffraction peak of MgO was not detected, which may be because the impregnation method caused the uniformly distribution of Mg (NO_3_)_2_·6H_2_O on the surface of ZrO_2_. MgO generated by pyrolysis after baking did not yet reach its detection threshold, so its diffraction peak was not detected in the XRD pattern.

#### 3.4.2. TEM characterization of MgO/ZrO_2_

**Fig. 12:**
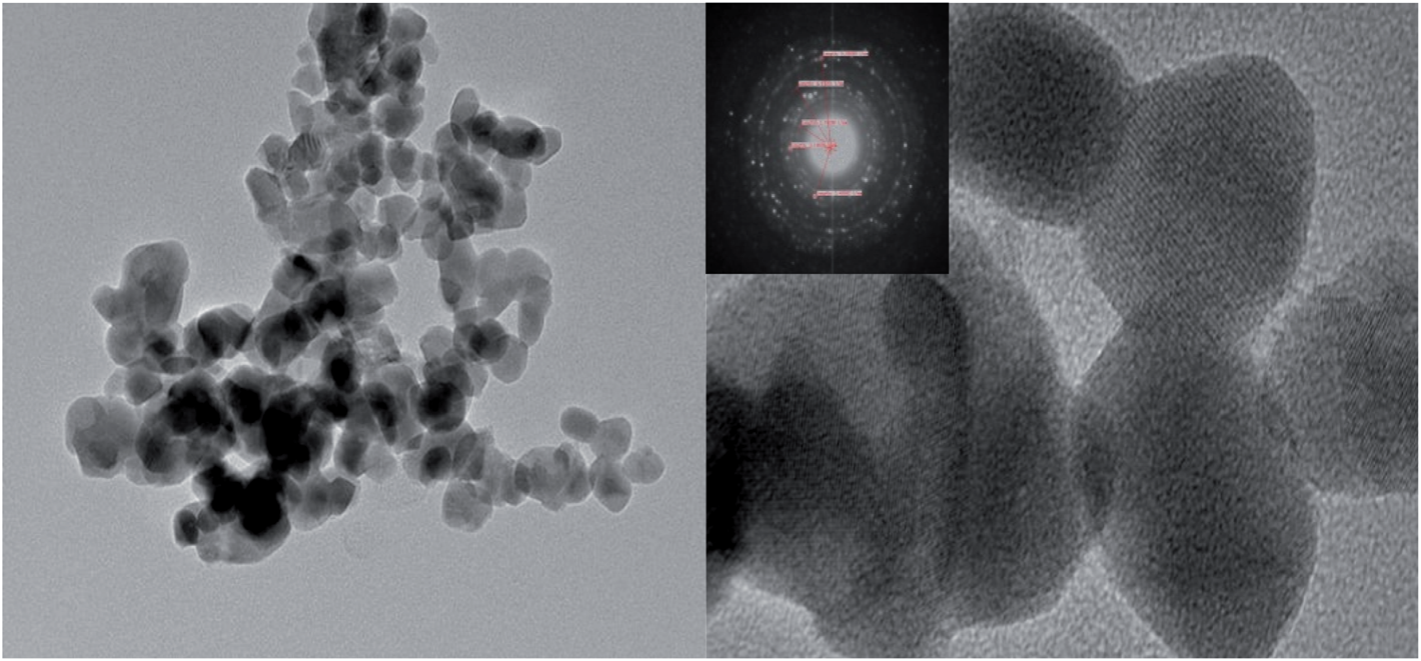
TEM characterization of the MgO/ZrO_2_ solid base prepared by impregnation at 700 °C for 6 h.

It can be seen from the TEM image of MgO/ZrO_2_ that when the calcination temperature was 700 °C and the calcination time was 6 h, the elements of the catalyst prepared by the impregnation method are evenly distributed, which indicates that Mg is highly dispersed in ZrO_2_ and mainly exists in the crystal form. The selected area diffraction pattern shows a concentric circle, which shows that the sample is polycrystalline, which is consistent with the XRD results (Fig. 12).

#### 3.4.3. N_2_ adsorption and desorption characterization of MgO/ZrO_2_

**Fig. 13:**
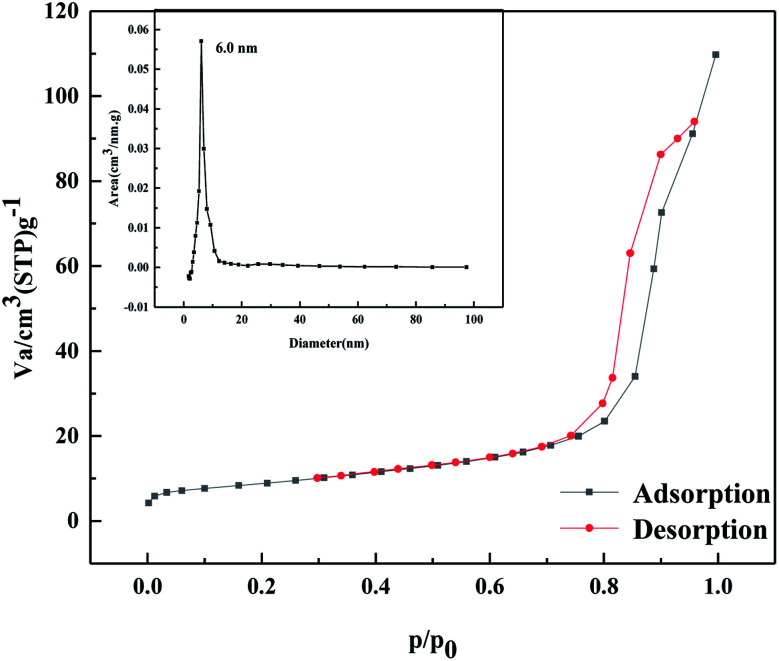
N_2_ adsorption–desorption curve of MgO/ZrO_2_ heat-treated at 700 °C.

As shown in Fig. 13, according to the Brunauer, Deming, Deming, and Teiler (BDDT) classification, the adsorption–desorption isotherm of the MgO/ZrO_2_ catalyst belongs to class IV, and it can be seen from the figure that the adsorption capacity gradually increases in the low-pressure section. At this time, N_2_ molecules are adsorbed on the inner surface of the mesoporous monolayer in a multi-layer. A sudden increase in the adsorption capacity at *P*/*P* = 0.75–0.9, forms a lag ring H1, which is the result of uniform pore agglomeration when the isotherm rises. The adsorption isotherm does not coincide with the desorption isotherm and the desorption isotherm is located above the adsorption isotherm, resulting in the adsorption lag phenomenon. It shows that the solid particles in the catalyst have a mesoporous structure. When the relative pressure is 0.75, the change in the slope of the isotherm is higher, indicating that the homogeneity of mesoporous materials is better.

#### 3.4.4. CO_2_-TPD diagram of MgO/ZrO_2_ prepared by the co-precipitation method and the impregnation method

**Fig. 14:**
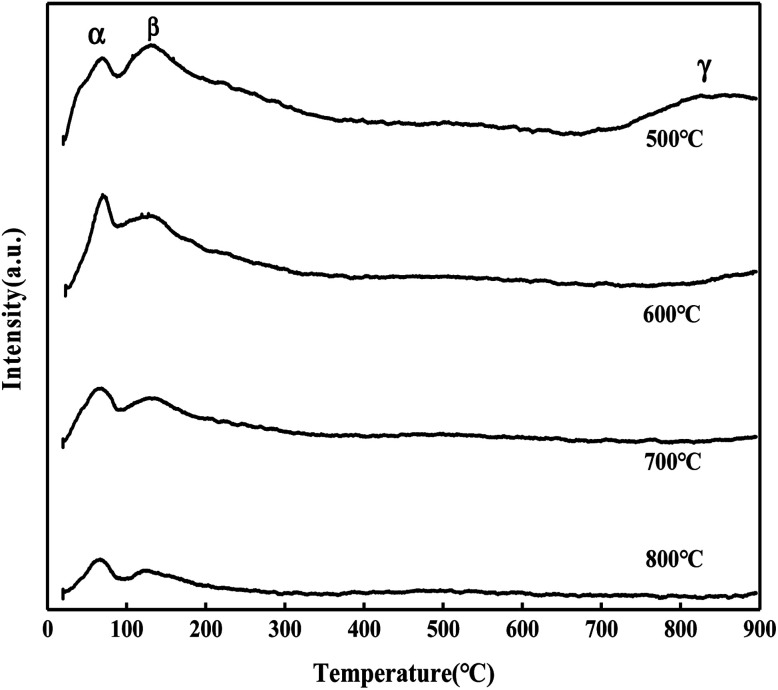
CO_2_-TPD diagram of MgO/ZrO_2_ prepared by the co-precipitation method.

According to Fig. 14, the CO_2_ desorption peaks of the MgO/ZrO_2_ catalyst can be divided into three types: α peak (50–100 °C), β peak (100–200 °C), and γ peak (750–900 °C). The α and β desorption peaks are similar to the CO_2_ desorption peak of ZrO_2_,^27^ which corresponds to the surface alkalinity of ZrO_2_. With the increase in the calcination temperature, the desorption amount of CO_2_ decreases significantly, and the position of the desorption peak slightly moves to the low temperature direction. The desorption temperature of the γ desorption peak is close to that of MgO, which can be attributed to the MgO desorption peak on the surface of ZrO_2_.

**Fig. 15:**
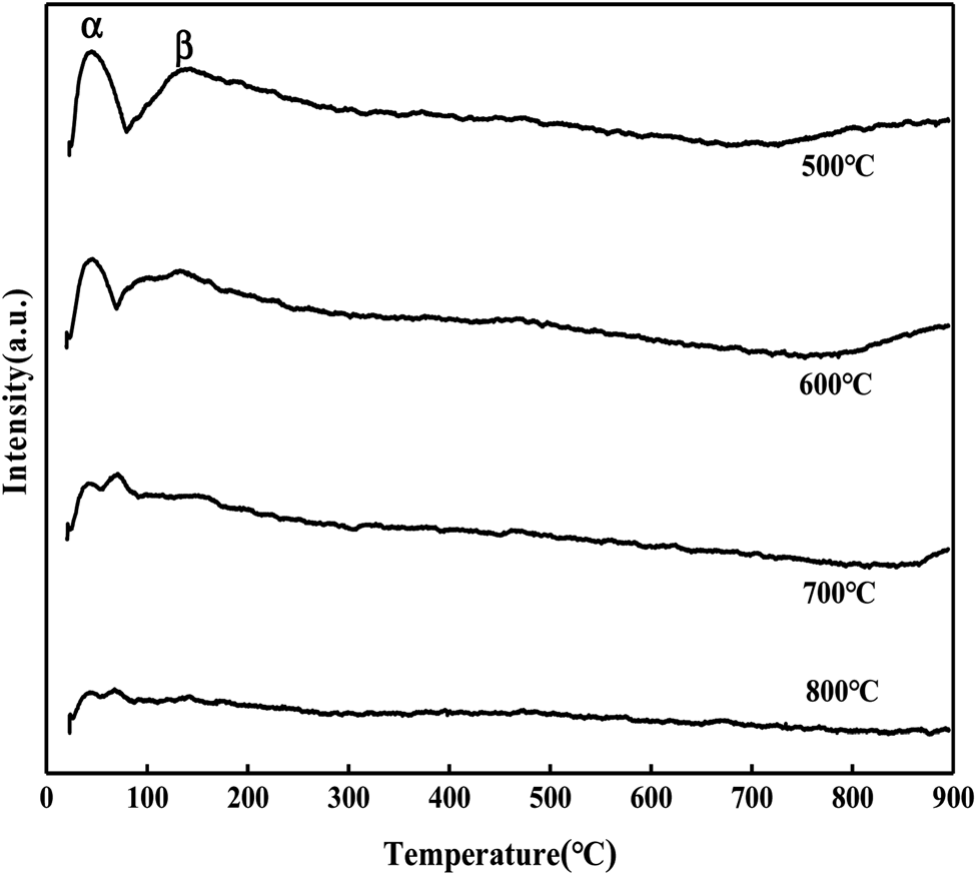
CO_2_-TPD diagram of MgO/ZrO_2_ prepared by the impregnation method.

It can be seen from Fig. 15 that the CO_2_ desorption peak of the MgO/ZrO_2_ catalyst prepared by the impregnation method is only the CO_2_ desorption peak of ZrO_2_, and the peak area and peak intensity are weak, and there is no γ desorption peak. With the increase in the calcination temperature, the desorption amount of CO_2_ is significantly reduced and less than the CO_2_ desorption amount of the MgO/ZrO_2_ catalyst prepared by the coprecipitation method. Also, compared with the coprecipitation method, the α and β desorption peaks in the impregnation method tend to move in the low temperature direction. Combined with XRD, the ZrO_2_ crystal phase is dominated by the monoclinic phase in the impregnation method and the tetragonal phase in the coprecipitation method. The basicity of tetragonal ZrO_2_ is slightly higher than that of the monoclinic phase. The stronger the basicity, the higher the catalytic activity, which has been verified in the above catalytic hydrolysis effect.

#### 3.4.5. XPS characterization of the MgO/ZrO_2_ solid base

**Fig. 16:**
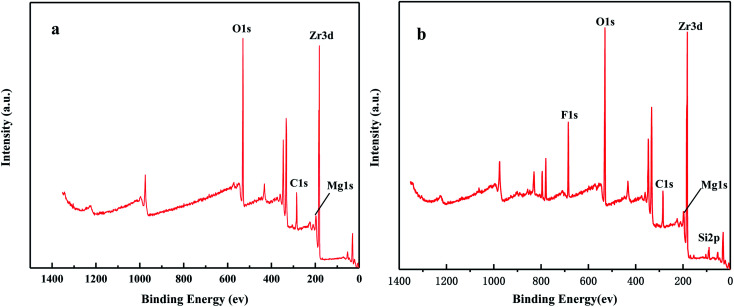
The full XPS spectrum of MgO/ZrO_2_ solid base before (a) and after (b) the reaction.

**Fig. 17:**
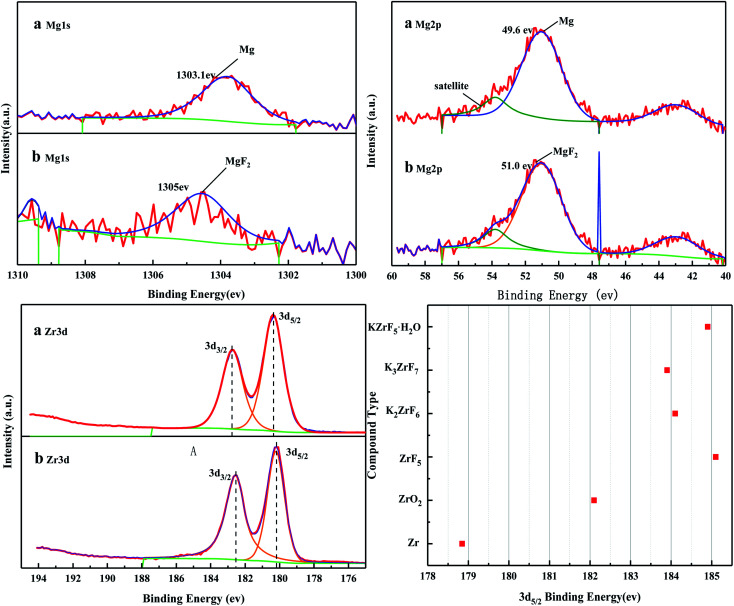
XPS fitting spectra of MgO/ZrO_2_ solid base before (a) and after (b) the reaction.

Fig. 16 shows the full XPS spectra before and after the MgO/ZrO_2_ reaction. It can be seen from the figure that new elements F and Si are introduced into the catalyst after the reaction. F is introduced by CFC-12 and Si is introduced by SiO_2_. By fitting the spectra of MgO/ZrO_2_ before and after the reaction, it was found that the Mg 1s spectra formed a main peak near 1303.1 eV and 1305 eV before and after the reaction. According to the X-ray photoelectron spectroscopy manual, it belongs to Mg and MgF_2_, respectively. Before and after the reaction, the main peak of Mg 2p is formed near 49.6 eV and 51.00 eV, which belongs to Mg and MgF_2_, respectively. For the Zr 3d spectrum in Fig. 17, there are two main peaks at 179.05 eV and 182.49 eV, as found out by consulting the Handbook of X-ray photoelectron spectroscopy, which belongs to the Zr and Zr^4+^ species. From the XPS parameters in Table 2 before the reaction, they mainly exist in the form of Mg and Zr, and after the reaction, they mainly exist in the form of Mg^2+^ and Zr^4+^, indicating that the oxidation reaction has taken place.

**Table 2:** XPS characterization of MgO/ZrO_2_ solid base before and after the reaction

Name	Pea BE	Areas (*P*) CPS. eV
a Zr3d_5/2_	179.05	106 203.83
Zr3d_3/2_	182.54	77 793.35
b Zr3d_5/2_	182.19	86 083.39
Zr3d_3/2_	184.53	85 421.70
a Mg1s	1303.1	2617.38
b Mg1s	1305	1487.31
a Mg2P	49.6	5644.51
b Mg2p	51.00	5916.44

#### 3.4.6. Effect on the catalyst preparation method on the conversion rate of difluorodichloromethane (CFC-12)

**Fig. 18:**
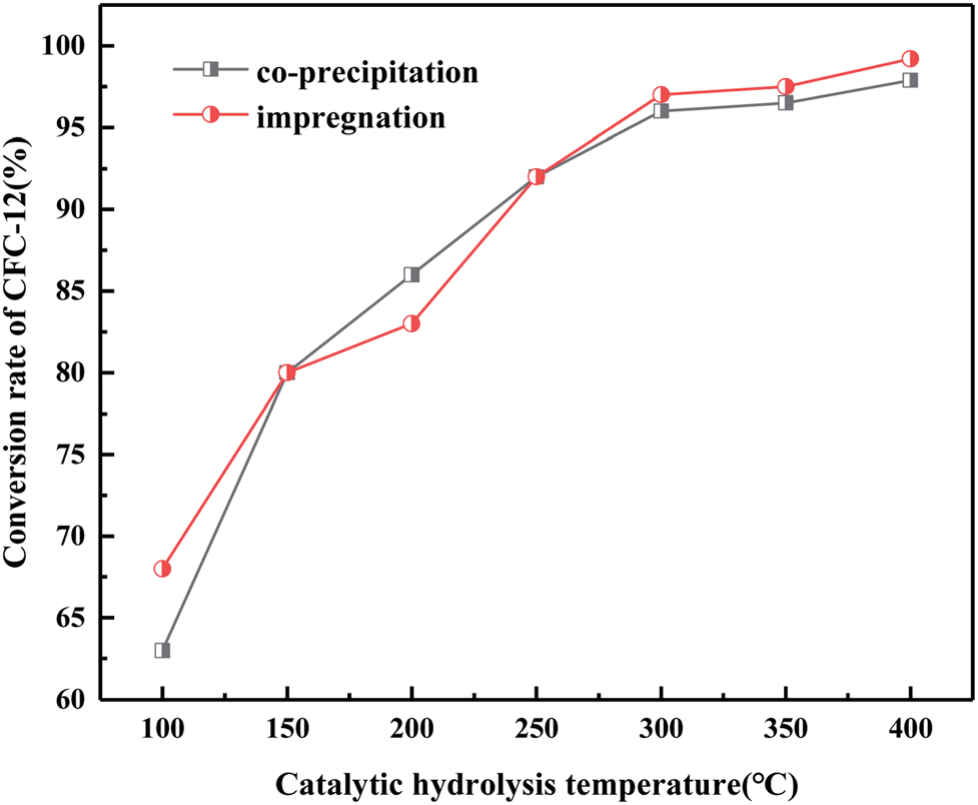
Effect of the catalyst preparation method on the conversion rate.

It can be seen from Fig. 18 that when the hydrolysis temperature is 300 °C, the conversion rate of CFC-12 prepared by the two methods is basically the same. The conversion rate of CFC-12 is 99.21% when the solid base MgO/ZrO_2_ catalyst is prepared by the impregnation method and the hydrolysis temperature is 300 °C. The conversion rate of CFC-12 is 98.13% when the solid base prepared by the co-precipitation method and hydrolysis temperature is 300 °C, which is only 1.08% different from that of the impregnation method. Research shows that the preparation method of the MgO/ZrO_2_ catalyst has little effect on the hydrolysis of CFC-12.

#### 3.4.7. Effect of MgO/ZrO_2_ prepared by co-precipitation and impregnation at different calcination temperatures on the conversion of CFC-12

**Fig. 19:**
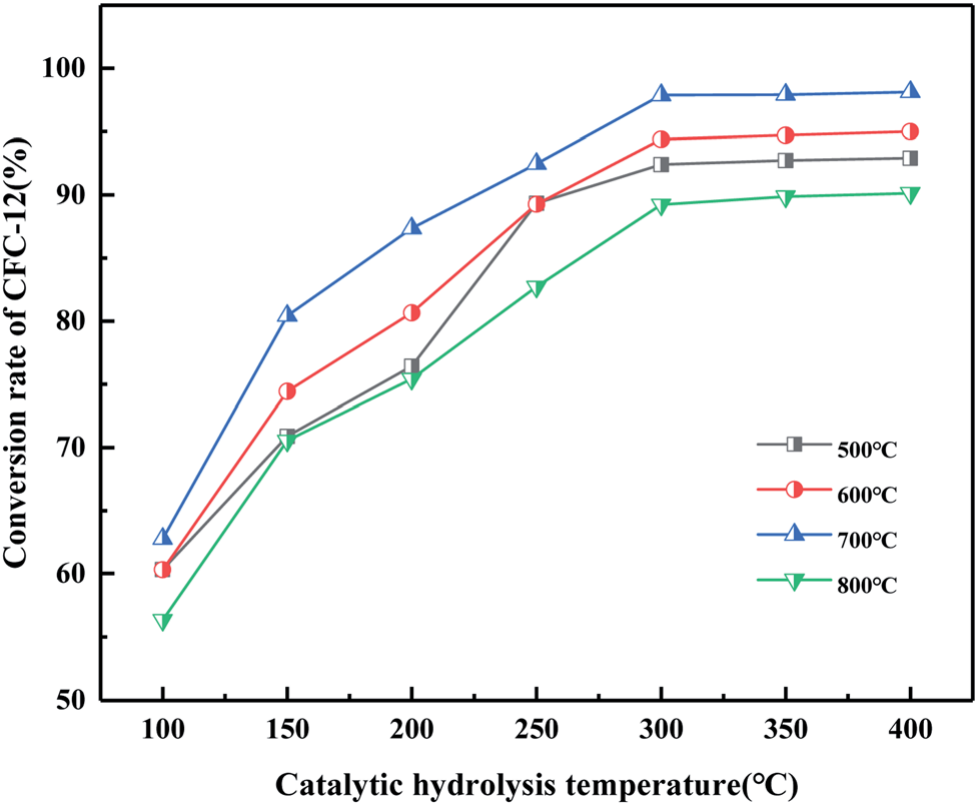
Effect of MgO/ZrO_2_ prepared by coprecipitation at different calcination temperatures on the conversion of CFC-12.

It can be seen from Fig. 19 that the catalyst calcined at 700 °C has the best catalytic hydrolysis effect on CFC-12. When the catalytic hydrolysis temperature is 400 °C, the conversion rate of CFC-12 reaches 98.13%. With the increase in the catalytic hydrolysis temperature, the conversion rate of CFC-12 gradually increases and the conversion rate of CFC-12 is basically stable when the hydrolysis temperature is in the range of 300–400 °C. Combined with the XRD and BET characterization results of the catalyst, when the calcination temperature is 700 °C, the ZrO_2_ diffraction peak of the catalyst is sharp with a moderate specific surface area and pore size, which shows a better catalytic hydrolysis effect, thus making the conversion rate of CFC-12 higher.

**Fig. 20:**
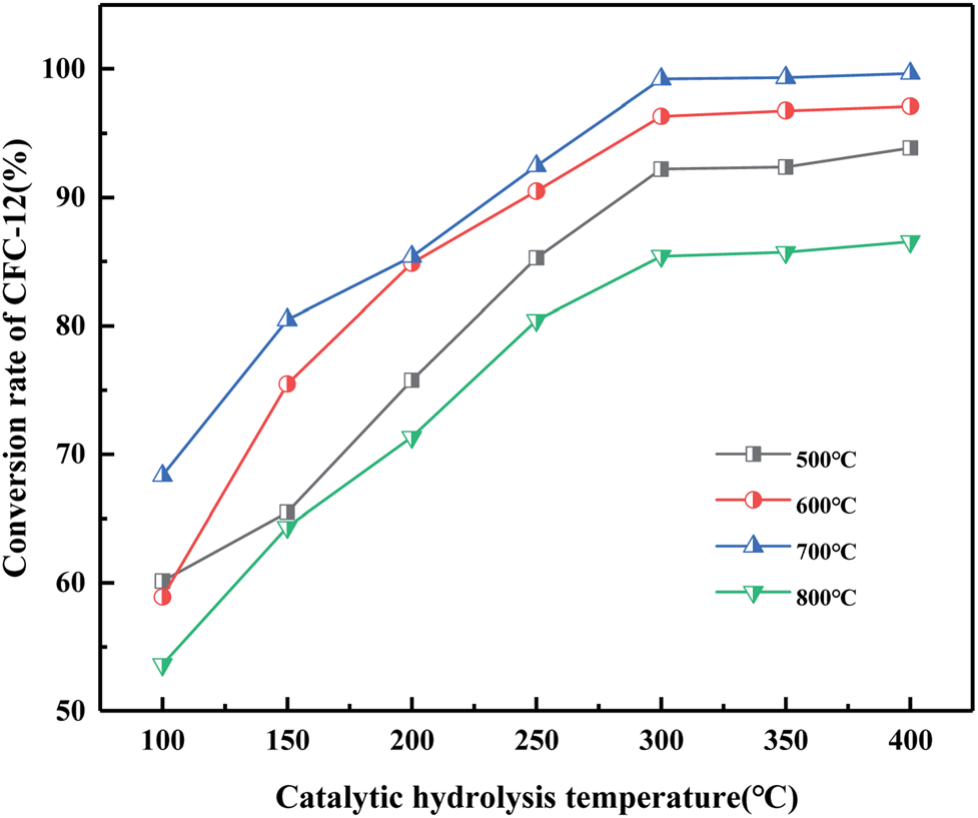
Effect of MgO/ZrO_2_ catalysts prepared by impregnation at different calcination temperatures on the conversion of CFC-12.

It can be seen from Fig. 20 that the trend of catalytic hydrolysis of CFC-12 is consistent with that of the MgO/ZrO_2_ catalyst prepared by the co-precipitation method on CFC-12. The conversion rate of CFC-12 was more than 99% when the calcination temperature was 700 °C and the catalytic hydrolysis temperature was 300 °C. With the increase in the calcination temperature, the hydrolysis rate decreased. It can be concluded that a very high or very low calcination temperature of the catalyst is not conducive for hydrolysis.

#### 3.4.8. Comparison of catalytic hydrolysis of CFC-12 by MgO, ZrO_2_, and MgO/ZrO_2_

**Fig. 21:**
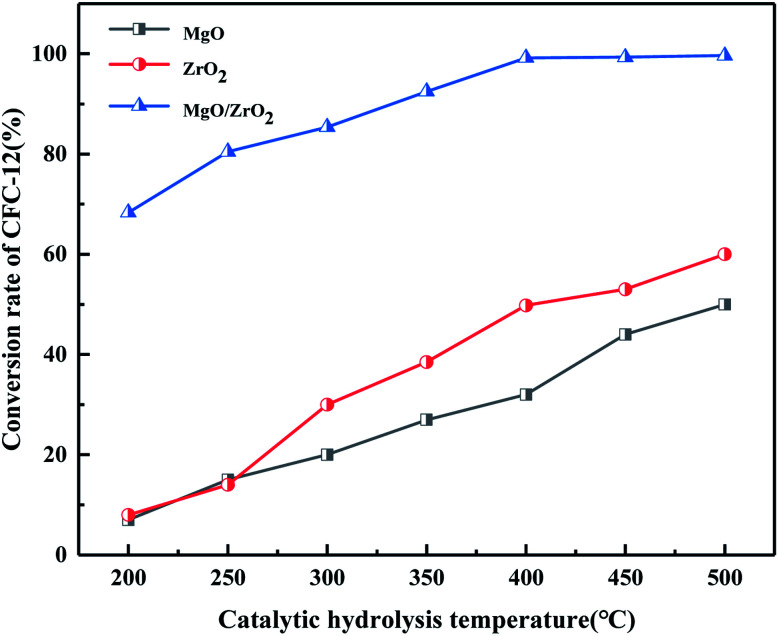
Comparison of catalytic hydrolysis of CFC-12 by MgO, ZrO_2_, and MgO/ZrO_2_.

It can be seen from Fig. 21 that MgO and ZrO_2_ have a certain catalytic activity for the hydrolysis of CFC-12, among which ZrO_2_ has a better catalytic activity; the conversion increased with the increase in temperature but the catalytic activity is only 59.63% at 500 °C. It could not reach our goal of efficient degradation of CFC-12. MgO/ZrO_2_ composites with MgO and ZrO_2_ had a high degradation rate of 99.21%; compared with MgO and ZrO_2_, MgO/ZrO_2_ is more efficient in the degradation of CFC-12.

### 3.5. BET characterization of solid base(acid) MgO (MoO_3_)/ZrO_2_

It can be seen from Table 3 that with the increase in the catalyst calcination temperature, the specific surface area and total pore volume of the catalyst gradually decrease, and, in turn, the average pore size increases. When the calcination temperature is 500 °C, the specific surface area of the catalyst prepared by the co-precipitation method is much higher than that by the impregnation method; the specific surface area is as high as 134.510 m^2^ g^−1^. As the calcination temperature increases, the specific surface area decreases sharply, indicating that the catalyst has a certain degree of sintering. Combined with XRD, the intensity of the diffraction peak is enhanced to a certain extent with the increase in the calcination temperature. The specific surface area of the catalyst calcined at 800 °C is reduced by 119.96 m^2^ g^−1^ compared with that at 500 °C. The surface area of the catalyst prepared by the impregnation method slowly decreases as the calcination temperature increases. Combined with XRD, with the increase in the calcination temperature, monoclinic ZrO_2_ is the main phase, the diffraction peak becomes sharp, the crystallinity increases, and the grain size increases. It can be seen that the ZrO_2_ crystal phase transitions and the catalyst surface is sintered, which causes the specific surface area of the catalyst to gradually decrease.

**Table 3:** Specific surface area, average pore diameter, and total pore volume of solid base(acid) MgO (MoO_3_)/ZrO_2_

	Calcination temperature	Specific surface area/m^2^ g^−1^	Average pore size/nm	Total pore volume/cm^3^ g^−1^
Coprecipitation MgO/ZrO_2_	500 °C	134	2.41	0.237
	600 °C	59	4.63	0.216
	700 °C	34	6.94	0.177
	800 °C	14	14.1	0.162
Dipping MgO/ZrO_2_	500 °C	85	3.55	0.232
	600 °C	51	6.06	0.197
	700 °C	30	6.06	0.170
	800 °C	20	9.21	0.167
MoO_3_/ZrO_2_	400 °C	245	1.66	0.115
	500 °C	197	2.41	0.221
	600 °C	95	2.31	0.136
	700 °C	63	3.12	0.125

### 3.6. Equivalent analysis of difluorodichloromethane (CFC-12) hydrolysis catalyzed by solid acid (base) MoO_3_(MgO)/ZrO_2_

It can be seen from Table 4 that the solid acid MoO_3_/ZrO_2_ was calcined at 600 °C for 3 h, the solid base MgO/ZrO_2_ (coprecipitated) was calcined at 700 °C for 6 h, and the solid base MgO/ZrO_2_ (impregnated) calcined at 700 °C for 6 h. When the catalytic hydrolysis temperature is in the range of 300–400 °C and the CFC-12 concentration is 4%, the catalytic hydrolysis products are CO, HCl, and HF, and the conversion rate of CFC-12 reaches 95–100%, indicating that the solid acid MoO_3_/ZrO_2_ and the solid alkaline MgO/ZrO_2_ catalytic hydrolysis of CFC-12 is equivalent. The phases of the solid acid MoO_3_/ZrO_2_ and solid base MgO/ZrO_2_ (co-precipitation) catalysts are mainly the tetragonal ZrO_2_ crystal phase. For the solid base MgO/ZrO_2_ (impregnation), the phase is a mixed state of tetragonal ZrO_2_ and monoclinic ZrO_2_, and both have a moderate specific surface area.

**Table 4:** Equivalence analysis of CFC-12 hydrolyzed by solid acid(base) MoO_3_(MgO)/ZrO_2_

Judging conditions		Solid acid MoO_3_/ZrO_2_	Solid base MgO/ZrO_2_ (coprecipitation)	Solid base MgO/ZrO_2_ (impregnation)
Catalyst preparation conditions	Calcination temperature	600 °C	700 °C	700 °C
	Calcination time	3 h	6 h	6 h
Catalytic conditions	Hydrolysis temperature	300–400 °C		
	CFC-12 concentration	4%		
	Hydrolysis rate	98.61%	97.89%	99.21%
	Catalytic hydrolysate	CO, HCl, HF		
Characterization results	Phase composition	*t*-ZrO_2_	*t*-ZrO_2_, *m*-ZrO_2_	
	Specific surface area	134 m^2^ g^−1^	34 m^2^ g^−1^	31 m^2^ g^−1^
	NH_3_/CO_2_ adsorption capacity	0.58476	0.63761	0.54632
	Acidity/basicity	Weak acidity	Weak alkalinity	Weak alkalinity

**Fig. 22:**
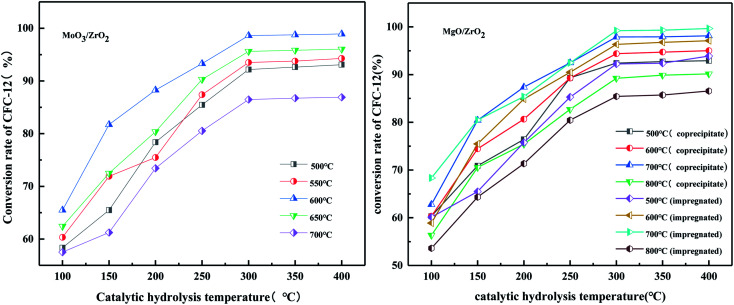
Catalytic hydrolysis of difluorodichloromethane (CFC-12) by solid acid MoO_3_/ZrO_2_ and solid base MgO/ZrO_2_.

The above research results show that the supported solid acid MoO_3_/ZrO_2_ and solid base MgO/ZrO_2_ catalysts have strong catalytic activity for the catalysis of CFC-12, and are equivalent to a certain extent. Taking the CFC-12 hydrolysis rate of 95–100% as the evaluation criterion, the catalyst preparation conditions (catalyst calcination temperature and calcination time), catalytic hydrolysis conditions (catalytic hydrolysis temperature and CFC-12 concentration), and catalytic hydrolysate combined catalyst characterization results (phase composition, specific surface area, and NH_3_/CO_2_ adsorption capacity) were comprehensively studied and analyzed, concluding the equivalence of CFC-12 catalyzed by solid acid MoO_3_/ZrO_2_ and solid base MgO/ZrO_2_ (Fig. 22).

## 4 Conclusions

The solid acid MoO_3_/ZrO_2_ and solid base MgO/ZrO_2_ catalysts respectively catalyze the hydrolysis of CFC-12. For the solid acid MoO_3_/ZrO_2_ catalyst calcined at 600 °C for 3 h, the CFC-12 hydrolysis rate is convenient when the catalytic hydrolysis temperature is 400 °C, which reached 98.91% decomposition. For the solid base MgO/ZrO_2_ catalyst roasted at 700 °C for 6 h by the co-precipitation method, the conversion rate of CFC-12 reached 98.13% at a catalytic hydrolysis temperature of 400 °C, and solid base roasted at 700 °C for 6 h by dipping method the MgO/ZrO_2_ catalyst has a CFC-12 conversion rate of 99.64% at a catalytic hydrolysis temperature of 400 °C. The solid acid MoO_3_/ZrO_2_ and solid base MgO/ZrO_2_ catalysts both exhibit very good catalytic activity when catalytically hydrolyzing CFC-12. The solid acid MoO_3_/ZrO_2_ and solid base MgO/ZrO_2_ catalysts have strong catalytic activity when catalyzing CFC-12, and are equivalent to a certain extent. For the solid acid MoO_3_/ZrO_2_ calcined at 600 °C for 3 h, the solid base MgO/ZrO_2_ calcined at 700 °C for 6 h (coprecipitation), and the solid base MgO/ZrO_2_ calcined at 700 °C for 6 h (impregnated) at a catalytic hydrolysis temperature in the range of 300–400 °C and CFC-12 concentration of 4%, the catalytic hydrolysis products are CO, HCl, and HF, and the CFC-12 hydrolysis rate reached 95–100%, indicating that solid acid MoO_3_/ZrO_2_ and solid base MgO/ZrO_2_ catalysis hydrolysis of CFC-12 is equivalent. The phases of the solid acid MoO_3_/ZrO_2_ and solid base MgO/ZrO_2_ catalysts are mainly the tetragonal ZrO_2_ crystal phases, and all have moderate specific surface areas.

## Abbreviations

CFC-12: Difluorodichloromethane
CFCs: Chlorofluorocarbon

## Author contributions

The manuscript was written by contributions of all authors.

## Funding sources

No competing financial interests have been declared.

## Conflicts of interest

There are no conflicts of interest.

## Supplementary Material
